# Selecting, implementing and evaluating patient-reported outcome measures for routine clinical use in cancer: the Cancer Care Ontario approach

**DOI:** 10.1186/s41687-020-00270-1

**Published:** 2020-11-26

**Authors:** Nicole Montgomery, Doris Howell, Zahra Ismail, Susan J. Bartlett, Michael Brundage, Denise Bryant-Lukosius, Monika Krzyzanowska, Lesley Moody, Claire Snyder, Lisa Barbera, The Cancer Care Ontario Patient Reported Outcome Advisory Committee, Lisa Barbera, Zahra Ismail, Doris Howell, Michael Brundage, Claire Snyder, Monika Krzyzanowska, Denise Bryant-Lukosius, Patricia Pottie, Lianne Dupras, Lesley Moody, Susan J. Bartlett, Monica Staley, Lorraine Martelli, Nicole Montgomery

**Affiliations:** 1 Cancer Care Ontario, Toronto, Canada; 2 University Health Network, Toronto, Canada; 3 University of Toronto, Toronto, Canada; 4 Women’s College Hospital, Toronto, Canada; 5 McGill University, Montreal, Canada; 6 McGill University Health Centre, Montreal, Canada; 7 Cancer Centre of Southeastern Ontario, Kingston, Canada; 8 Queens University, Kingston, Canada; 9 McMaster University, Hamilton, Canada; 10 Johns Hopkins University, Baltimore, USA; 11 Tom Baker Cancer Centre, Calgary, Canada; 12 Cancer Control Alberta, Edmonton, Canada

**Keywords:** Patient-reported outcome measures, Clinical care, Routine care, Recommendations, Symptom management

## Abstract

**Background:**

The use of Patient-Reported Outcome Measures (PROMs) in routine clinical care can help ensure symptoms are identified, acknowledged and addressed. In 2007, the provincial cancer agency, Cancer Care Ontario, began to implement routine symptom screening with the Edmonton Symptom Assessment System (ESAS) for ambulatory cancer patients. Having had a decade of experience with ESAS, the program developed a strategic interest in implementing new and/or additional measures. This article describes the development of a streamlined PROM selection and implementation evaluation process with core considerations.

**Methods:**

Development of the PROM selection and implementation evaluation process involved analysis of quantitative and qualitative data as well as consensus building through a multi-stakeholder workshop. Core PROM selection considerations were developed through a literature scan, review and refinement by a panel of methodological experts and patient advisors, and testing via a test case. Core PROM implementation evaluation considerations were developed through analysis of PROM evaluation frameworks, and review and refinement by a committee of provincial implementation leads.

**Results:**

Core PROM selection considerations were identified under three overarching themes: symptom coverage, usability and psychometric properties. The symptom coverage category assesses each PROM to determine how well the PROM items address the most prevalent and burdensome symptoms in the target patient population. The usability category aims to assess each measure on characteristics key to successful implementation in the clinical setting. The psychometric properties category assesses each PROM to ensure the data collected is credible, meaningful and interpretable. A scoring system was developed to rate PROM performance by assigning a grade of “weak”, “average” or “good” for each category. The process results in a summary matrix which illustrates the overall assessment of each PROM. Implementation evaluation considerations were identified under three overarching concepts: acceptability, outcomes, and sustainability. A consensus building exercise resulted in the further identification of patient, provider, and clinic specific indicators for each consideration.

**Conclusion:**

To address the need for a systematic, evidence-based approach to selection, implementation and evaluation of PROMs in the clinical setting, Cancer Care Ontario defined a process with embedded core considerations to facilitate decision-making and encourage standardization.

## Introduction

The Canadian Cancer Society (2019) estimates that half of all Canadians will develop cancer in their lifetime, and about a quarter will die from it. In 2019 alone, it is estimated that 220,400 Canadians will be diagnosed with cancer and 82,100 will die from the disease [1]. Cancer and its treatment can cause significant physical and emotional distress [2] which if left unaddressed can lead to diminished quality of life [3–5]. Poorly managed, these problems can be costly to the health system [6, 7]. For example, in Ontario 40% of breast cancer patients undergoing adjuvant treatment visit the emergency department (ED) within the first 2 months of treatment [6]. Although, many factors can lead to ED use, lack of systematic standardized assessment of symptoms leading to inadequate symptom management [8, 9], and poor patient/clinician communication regarding patients’ symptoms [10] are contributors. In fact, without a standardized tool, most patients’ distressing/bothersome symptoms may never be discussed in clinic visits [11] and more than half of patients may never receive adequate care for their symptoms [12].

Patient-Reported Outcome Measures (PROMs) in routine clinical care can help ensure symptoms are identified and addressed [12] A PROM is a validated measure that provides the patient’s perspective on disease symptoms, treatment side effects, functional status, well-being, and/or quality of life (QOL). The implementation of routine symptom screening using tailored PROMs can improve patient/provider communication, help to monitor treatment response, and identify unrecognized problems [10].

In 2007, the provincial cancer agency (Cancer Care Ontario, CCO) implemented routine electronic symptom screening at a system-level in the ambulatory oncology setting. The cancer agency developed an electronic platform called the Interactive Symptom Assessment and Collection tool. Of the 74 hospitals that provide cancer care in Ontario, 64 are currently collecting PROMs using this electronic platform. In Ontario, over 40,000 symptom screens from cancer patients are collected each month, making Cancer Care Ontario’s database one of the largest patient-reported outcomes repositories in the world [13]. The Edmonton Symptom Assessment System Revised (ESAS-r), a validated and reliable PROM with utility across cancer populations, is the PROM routinely collected [13]. In 2013, the patient-reported Eastern Cooperative Oncology Group performance status tool (pECOG) was added.

This large-scale, standardized symptom screening program provides a unique opportunity to understand the impact of cancer and accelerate the use of PROMs in clinical care. Beyond implementing the ESAS and pECOG, the provincial cancer agency aims to systematically collect PROM data to trigger the assessment of symptoms that are most relevant to specific cancer populations. For example, province-wide implementation of a PROM for prostate cancer patients, the Expanded Prostate Cancer Index – Clinical Practice (EPIC-CP), was completed in June 2018 following a pilot test of implementation [14].

As the program continues to mature, a structured governance framework has been established to guide the program’s strategic vision and priorities. Specifically, a PROs Advisory Committee (PROs-AC) was convened and includes methodological and clinical experts as well as patient advisors. The objective of the committee is to guide the provincial cancer agency on the prioritization, identification, selection, implementation and evaluation of disease-agnostic (e.g., ESAS) and disease-specific PROMs for cancer patients. A PROs-implementation collaborative made up of local leads (clinicians, clinic managers, and champions) from each of the 14 Regional Cancer Programs, and Patient and Family Advisors acts as the implementation arm to ensure successful and sustainable uptake of PROMs within the cancer programs.

A streamlined process to guide selection, implementation and evaluation of PROMs is critical to realize the maximum impact and ensure sustainability of these large-scale complex projects. However, little guidance is available to help organizations make PROM selection and implementation decisions while accounting for competing selection criteria. Consequently, the provincial cancer agency through the expertise of the PROs-implementation collaborative and PROs-AC developed a PROM selection and implementation process with considerations specifically to promote use and sustainability in clinical care. This paper describes how the PROM selection and implementation evaluation processes were developed and shares an example of how it works using a test case so it can be leveraged by others.

## Methods & materials

### PROM selection process

Development of the PROM selection process began with a search of the peer reviewed literature, guidelines, and other grey literature to identify existing resources related to PROM selection. Search terms such as Patient Reported Outcome, PROMs, selection, implementation guidelines, criteria, and recommendations were used. Resources related to the use of PROMs in routine clinical care, and secondarily use of PROMs in the research setting, were included. The most relevant resources determined by applicability to the clinical setting and usefulness for decision-making were used to compile a list of potential selection considerations that were grouped by category and ranked by number of citations in the literature. Through expert consensus, the committee used the common themes as categories, and defined core considerations for each category.

A test case was used to determine if the established selection process and core considerations facilitated the selection of a PROM for head and neck cancer. The PROs-AC convened for a half-day consensus meeting to discuss the test case selection considerations, evaluate the six candidate PROMs, and select the best performing measure using the selection criteria.

### PROM implementation evaluation process

The implementation process was developed by compiling, ranking, and refining > 75 candidate considerations used in past PROM pilot evaluations, identified in the literature. The PROs-AC met over the course of 6 months to review the list of potential considerations and determine the importance of each consideration for rigorous and meaningful PROM implementation evaluation. The expert team was also instructed to indicate if there were any missing considerations (based on their experience and expertise). Lastly, the PROs-implementation collaborative was convened to carry out a consensus building exercise on the list of considerations, which involved presentations, real-time group feedback, and consensus by the group of ~ 30 stakeholders.

## Results

### PROM selection process

The literature scan resulted in 25 relevant articles related to the PROM selection process. Abstract review identified the six documents from which three common themes related to PROM selection emerged: **Symptom Coverage, Usability** and **Psychometric Properties**. The documents included: (1) Pan-Canadian Oncology Drug review (pCODR) Deliberative Framework [15]; (2) Agency of Clinical Innovation NSW - Integrated Care: Patient reported outcome measures and patient reported experience measures – a rapid scoping review [16]; (3) User’s Guide to Implementing Patient-Reported Outcomes Assessment in Clinical Practice. ISOQOL 2011 [17]; (4) ISOQOL recommendations for minimum standards for patient-reported outcome measures used in patient-centered outcomes and comparative effectiveness research [18]; (5) Boyce, Browne, & Greenhalgh, 2014. Experience of professionals using information from patient-reported outcome measures to improve the quality of healthcare: a systematic review of qualitative research [19]; and (6) COSMIN Manual for Systematic Reviews of PROMs [20].

#### Symptom coverage considerations

The objective of the symptom coverage category as determined through the literature search as well as the PROs-AC, is to assess the relevance of each candidate PROM for the target population and determine which PROM(s) best address the symptoms that matter most to those patients. Therefore, the main consideration for symptom coverage is that the measure address the most relevant symptoms for the target cancer (i.e., the symptom list changes depending on the target patient population). The symptoms should be identified through a literature review (evidence-based). Each symptom is then categorized as: 1) prevalent, 2) prevalent and burdensome, or 3) a critical consideration, which means it had been endorsed by clinical experts as a “must have” symptom. This categorization facilitates further evaluation to ensure coverage of the most important symptoms. This step is completed with input from the relevant provincial tumor team and allied health team members (e.g. dietician). Each candidate PROM is inventoried to determine how many relevant symptoms are addressed in the measure, and how many items exist per symptom.

#### Usability considerations

The usability category required extensive input from experts as there were only a few mentions of usability considerations identified within the literature. For example, the PCORI Methodological Minimum Standards Paper identifies 12 concepts as minimum standards for the development, selection, and use of patient reported outcomes data in patient centred outcome research, only two of which were deemed appropriate for usability considerations - symptom burden and appropriate recall time. The usability category ultimately included the following core considerations: [1] conceptual characteristics (such as number of items, type of scale, and recall timeframe), [2] scoring (domain, item, and/or global scores), [3] time to complete the measure, [4] use of plain language [5] available translations (how many available languages), and [6] licensing or fees for use.

#### Psychometric property considerations

In contrast to the usability category, there was substantial evidence to guide the selection of key psychometric properties. For example, the PCORI minimum standards includes 8 concepts related to psychometrics properties reliability, interpretation of meaningful change, content validity, sampling in PROM development, construct validity, ability to detect change, modification of existing PROM, and establishing multi-mode equivalence. Therefore, the PROs-AC used their own judgement and consensus building to select those considerations from the literature they felt were most important and most relevant to the clinical context. The final list of psychometric core considerations included: [1] internal consistency, [2] test-re-test reliability, [3] responsiveness, [4] discrimination ability, [5] meaningful change, and [6] translation validity.

A **Summary Matrix** is used at the end of the process to illustrate the overall performance of each measure. Initially the PROs-AC tried to assign a numerical score to each category, with the objective of assigning an overall score to each PROM. However, it was quickly determined that a numerical score did not capture the nuance in each category, and it was more appropriate to assign a grade. The PROs-AC decided to assign grades of “weak”, “average” or “good” for each category (Symptom Coverage, Usability, Psychometric Properties). The PROs-AC also decided on critical considerations under each category that were essential and should be required for the PROM to be useful in the clinical setting. For example, under the usability category is the consideration that the PROM should have less than 30 items because more items would be too long for routine clinical use. If any of these critical considerations are not met, the committee decided an “X” should be placed in the category.

**Fig. 1:**
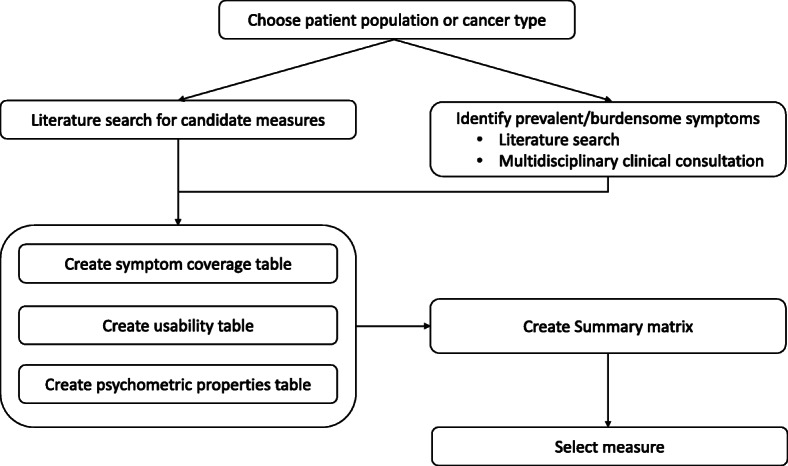
Provides an overview of the steps in the PROM selection process

An overall summary of the application of the selection process is described in Fig. 1.

### PROM implementation evaluation process

Table 1 shows the standardized PROM implementation evaluation process and core considerations. The PROs AC identified 10 concepts that should be considered when implementing and evaluating a PROM pilot, and grouped them under 3 categories **acceptability, outcomes**, and **sustainability**. The PROS-implementation collaborative further identified the need to account for multiple stakeholders’ experiences when considering the success or failure of a PROM pilot. Ultimately, each core consideration is further broken down to include evaluation from the patient, provider, and clinic perspectives. The implementation evaluation process can be used to guide uptake and evaluate PROM pilots to ensure each one is assessed according to these minimum standards before being considered for spread and scale across a jurisdiction.

**Table 1:** PROM implementation evaluation process and core considerations

	Consideration	Patient	Provider	Clinic
**Acceptability**	**Relevance/Importance**	Patients feel the PROM addresses symptoms that are relevant and important	Providers feel results of the PROM are clinically relevant and amenable to change	The PROM facilitates collection of clinic level data to better understand symptom burden and need for supportive care
	**Interpretability**	Patients feel the PROM and symptom report are easy to understand/interpret	Providers feel the PROM scores and symptom report are easy to interpret	The PROM facilitates real-time bi-directional flow of information between patients and care providers
	**Clinic Flow Integration**	Patients feel the PROM is easy to complete (not too long)	Providers feel the PROM is concise (enough time to address scores)	The PROM can be easily integrated in clinic flow and completed < 11 min
**Outcomes**	**Communication**	Patients feel PROM helps them communicate their symptoms to their care providers	Providers feel the PROM helps facilitate discussion with patients about their symptoms	The PROM facilitates communication between providers (within and between disciplines)
	**Symptom Recognition**	Patients feel their PROM scores help providers acknowledge their symptoms of concern	Providers feel the PROM scores help to ensure symptoms of concern are recognized	The PROM facilitates shared understanding and decision-making between patients and providers
	**Focused Symptom Assessment**	Patients feel the PROM helps providers focus and track symptoms that matter most to them	Providers feel the PROM scores help focus the visit on symptoms of concern	The PROM facilitates efficiency by focusing visit time on symptoms of most concern/severity
	**Appropriate intervention/Referral**	Patients feel the PROM scores help ensure their most bothersome symptoms are being addressed	Providers feel the PROM scores help ensure the most bothersome symptoms have been addressed	The PROM facilitates appropriate intervention (triage/referrals)
	**Perceived Value-add**	Patients feel the PROM adds to their care and is worth completing	Providers feel the PROM adds value to their practice	The PROM is perceived to be a valuable component of patient care throughout the clinic (clinic culture)
**Sustainability**	**Potential for Embedding in Routine Practice**	Patients feel completing the PROM is a routine part of their clinic visit	Providers feel addressing PROM scores is a routine part of the clinic visit (and their practice)	The PROM fits in the model of care, has become normalized within the clinic operations, and is supported by leadership
	**Support and Resources**	Patients feel there is sufficient support to complete the PROM (volunteers, technology, patient symptom guides)	Providers feel there is sufficient resources to support PROM use (staff, hardware, education, symptom guides etc.)	Routine PROM completion is a priority within the clinic (allocated sufficient human and technical resources)

#### Acceptability core considerations

The acceptability category aims to address the extent to which the PROM worked well for patients and providers as well in the clinic as a whole. It is extremely important to assess acceptability from these three perspectives as there are measures that providers dislike because they feel they can do nothing in response. However, patients are happy to complete those same questions when the symptoms are relevant to their specific needs and are being recognized and normalized through completion of the PROM, and acknowledgement by the provider [14]. The acceptably considerations include relevance/importance, interpretability, and clinic flow integration.

#### Outcome core considerations

Outcomes are very important in determining the extent to which any changes/improvements in care where observed throughout the pilot, as many of these considerations can in the long term affect patients’ health outcomes. The outcome considerations include communication, symptom recognition, focused symptom assessment, appropriate intervention/referral, and perceived value-add.

#### Sustainability core considerations

The sustainability category aims to address the degree to which it is feasible to continue using the PROM. This concept is also very important in resource planning when considering whether to expand implementations. Sustainability considerations include potential for embeddedness in routine clinical practice and support and resources.

## Application to a head and neck test case

Literature review was conducted to identify candidate measures. Details of this review are summarized in a separate manuscript (under review). Six candidate measures were identified.

Table 2 shows an example of a completed symptom coverage table from the head and neck cancer test case. Completion of the table successfully facilitates a meaningful comparison of the head and neck candidate PROMs’ strengths and weaknesses and overall symptom coverage.

**Table 2:** PROM selection process symptom coverage category – head and neck test case

Symptom (each cell shows # of items for each symptom)	EORTC HN	FACT HN	FHNSI-10	MDASI-HN	FHNSI-22	Vanderbilt
Swallowing difficulty or pain^b^	**4**	**2**	**2**	**2**	**1**	**8**
Saliva function^a^	**2**	**1**		**2**		**11**
Jaw mobility or jaw pain	**2**					**1**
Chewing/Teeth problems^b^	**1**			**2**	**2**	**7**
Taste (smell) changes^a^	**2**			**1**	**1**	**4**
Appetite/weight change^a^	**2**			**1**	**2**	**3**
Mouth or throat pain^b^	**3**	**1**	**1**	**1**	**2**	**7**
Pain (overall)^a^	**1**	**1**	**1**	**1**	**1**	**4**
Skin issues				**1**		
Shortness of breath or coughing	**1**	**1**	**1**	**1**	**1**	
Hoarseness (while talking)^b^	**3**	**2**	**1**	**1**	**1**	**3**
Hearing loss						**1**
Depression		**3**		**3**	**1**	
Anxiety^a^		**3**	**1**		**1**	
Body image^a^	**1**	**1**			**1**	
Substance abuse						
Social interactions difficulties	**4**	**3**		**1**	**1**	
Sexual functioning^a^	**2**	**1**				
Fatigue^a^		**2**	**1**	**1**	**1**	
Drowsiness						
Sleep quality^a^		**1**		**1**	**1**	
**Total Symptom Coverage Score**	**Average**	**Average** **X chewing**	**Weak** **X chewing**	**Good**	**Good**	**Average**

**Table Legend**

Prevalent

^a^Prevalent and burdensome

^b^Critical consideration

**Scoring Legend**

**Weak**: ≤ 33%

**Average**: 34 - 65%

**Good**: ≥ 66%

**X**: Missing critical consideration

Table 3 shows the summary matrix from the head and neck test case. Each category has its own scoring legend to assign grades of weak, average, or good for that particular category. After evaluating all candidate measures in all 3 categories (symptom coverage, usability, and psychometric properties) a summary matrix is created. The test case successfully facilitated the identification of two PROMs that outperformed the rest. In the Symptom Coverage Category 2/6 candidate measures were rated as “good” (≥66% symptom coverage); 3/6 “average” (34% - 65% symptom coverage), with 1 receiving an X for not including a critical symptom (chewing); and 1 “weak” (≤33% symptom coverage) with an X for not including chewing. In the Usability Category, 5 measures scored “good” satisfying > 75% of the considerations, but 2 received an X for having more than 30 items. Lastly 1 measure scored “weak” for satisfying only 2/6 considerations, and also received an X for failing to meet the core consideration of < 30 items. In the Psychometric category 3/6 measures were rated “good” and 3 “average”. No measure received an X. Ultimately 2 measures (MDASI-HN and FHNSI-22 in Table 3) stood out as being good candidates. MDASI-HN was scored the best across all categories. However, FHNSI-22 received good on 2 of the 3 categories and was regarded by the group as being a strong candidate measure, warranting further consideration.

**Table 3:** PROM selection process summary matrix – head and neck test case

Summary Matrix						
	EORTC HN	FACT HN	FNHSI-10	MDASI HN	FHNSI-22	Vanderbilt
**Symptom Coverage**	Average	Average (X)	Weak (X)	Good	Good	Average
**Usability**	Good (X)	Good (X)	Good	Good	Good	Weak (X)
**Psychometric Properties**	Good	Good	Average	Good	Average	Average

Through completion of the head and neck test case it became clear that there are elements of each PROM such as wording, phrasing, and formatting that can only be evaluated from the perspective of patients. Although we had patient or family advisors as standing members of PROs-AC they did not have experience with head and neck cancer. Therefore, we adapted our selection plan to include a final step in the test case with focus groups involving patients with head and neck cancer. The 2 best preforming candidate measures as identified through the PROM Selection Process test case were shared with 22 patients with head and neck cancer. They completed the measures and provide feedback on their preferences. This exercise resulted in the selection of the PROM that met almost all of the core considerations and was overwhelmingly preferred by patients with head and neck cancer, highlighting the critical importance of patient engagement as a key component of PROM selection.

At the time of publication the pilot testing for the selected measure is ongoing. Data collection is via a combination of surveys, interviews and chart audit.

## Discussion

The PROM selection and implementation evaluation process with core considerations described in this paper provide comprehensive, expert-endorsed guidance to support the utilization of PROMs in clinical practice. The process was developed to ensure the same minimum standards are met across every PROM selection and implementation evaluation, while also considering the need to be flexible and allow for diverse settings and case specific needs. Patients experience many symptoms throughout their cancer journey. Poorly managed, these problems can have a major negative impact on quality of life and can be costly to the health system [3–7]. Using PROMs in clinical care can help ensure symptoms are identified and addressed [10, 11].

Despite the growing utilization of PROMs in clinical settings, the majority of guidance and recommendation documents initially identified through the literature search were designed with a focus on clinical trials, with only six documents deemed relevant for clinical care [15–20]. Though these six documents provided valuable insight, they would not be sufficient to guide the selection and implementation of PROMs for routine clinical care. We identified many articles suggesting that PROMs are a growing area of research in many parts of the world. Several articles reported successes of small and large-scale PROM implementations in the clinical setting [10]. The literature search reaffirmed the need for a guidance document or decision-making tool specifically designed to ensure PROMs implemented in clinical care meet specific requirements critical to uptake and sustainability in the clinical setting. Our work is meant to help address this gap.

The expanded and targeted use of PROMs in clinical practice is complex and therefore vulnerable to poor implementation, creating ‘busy-work’ without bringing meaningful change to the patient encounter. Failed implementation of health care innovations can waste resources and potentially impact patient care [21]. Conversely, when implementation is successful, it can affect whole systems or services, improving practice and optimizing patient care [21]. One of the goals of this development work was to be transparent and rigorous, and to engage stakeholders broadly. Engagement of key stakeholders (clinicians, patients, managers) throughout the process was essential to improving the likelihood of PROMs uptake in routine clinical care. Indeed, the rigor of this process has resulted in the measure selected from our head and neck cancer test case being adopted in other provinces (personal communication) and endorsed nationally [22].

The use of decision-making models, such as this PROM selection process, have proven valuable in other areas of healthcare. An example is the deliberative framework that the pan-Canadian Oncology Drug Review (pCODR) uses to make funding recommendation that considers clinical benefit, economic evaluation, adoption feasibility, and patient values [23]. pCODR has been using this decision-making process since its inception in 2011 and it has guided hundreds of funding recommendations that are nationally accepted. A streamlined process for PROM selection could similarly guide many decisions and encourage consistency in how PROMs are selected for implementation, measured, and compared across jurisdictions. Both the pCODR framework and the PROM selection process recognize the need for meaningful patient engagement as an input to decision-making.

The development of this process was successful because of the considerable infrastructure that existed within the provincial cancer agency during the time of the work. The provincial program structure, with a physician lead and dedicated staff, was a key enabler. The ability to draw on local, regional, and international expertise to create a strong PROs-AC in combination with a collaborative of local implementation leads, created a strong programmatic structure. Many organizations may not have access to similar resources, and we hope that sharing this developed process may facilitate high quality PROM work. Even with this process described, resources to apply this approach are still required. This includes the ability to conduct a literature search, to catalogue existing measures and to provide oversight for a process that relies on clinical experts, methods experts, operational team members and patients. While resources are required for a rigorous process, the payoff is a well vetted measure that will suit the needs of patients and the organization which can strengthen implementation; however, other approaches, which may be less resource-intense have been used in other jurisdictions [24].

This PROM Selection and Implementation Evaluation Process is usable in any jurisdiction but is not a stand-alone tool. Successful facilitation will still require engagement of multiple stakeholder groups. Evaluating candidate PROMs requires expertise and should not be applied without oversight. A multi-disciplinary group is needed to discuss such elements as clinical utility and burden versus research potential. Many of the considerations are subjective and require discussion with methodologists, clinicians, operational leaders, and patients. PROs-AC was able to reach consensus on most discussion points. This was facilitate by early decisions regarding programmatic goals. For example, we had agreed that we would use a modular approach where an entire measure is selected for a particular patient population, rather than individual items for individual symptoms. Also, we decided we would choose measures for a handful of cancer types but not every single last one. Furthermore, through the test case for head and neck cancer, the provincial cancer agency discovered that the engagement of patients with experience in the specific disease site/symptom area is fundamental to successful PROM selection. Finally, the approach was designed to be applicable for any other cancer type and provincial cancer agency has since used this approach to identify a measure for cervix cancer.

## Conclusion

To address a gap in available guidance documents and tools for the selection of PROMs and evaluation of PROM implementations in the clinic setting, provincial cancer agency defined a process with core considerations to facilitate decision-making and encourage standardization. Future directions include expanding PROs-AC to be national in scope and use the PROMs selection and implementation evaluation process to implement new PROMs in Ontario and other jurisdictions.

## Data Availability

Not applicable.

## Abbreviations

PROMs: Patient-Reported Outcome Measures
PROs-AC: PROs- Advisory Committee
QOL: Quality of life
ESAS-r: Edmonton Symptom Assessment System Revised
pECOG: Patient-reported Eastern Cooperative Oncology Group performance status tool
EPIC-CP: Expanded Prostate Cancer Index – Clinical Practice
